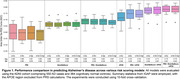# BrainNetScore: Enhancing Alzheimer’s disease risk prediction using genetic‐guided brain volumetric phenotype network

**DOI:** 10.1002/alz.084351

**Published:** 2025-01-03

**Authors:** Yonghyun Nam, Jakob Woerner, Sang‐Hyuk Jung, Erica H Suh, Haochang Shou, Li Shen, Dokyoon Kim

**Affiliations:** ^1^ Perelman School of Medicine, University of Pennsylvania, Philadelphia, PA USA; ^2^ University of Pennsylvania, Philadelphia, PA USA

## Abstract

**Background:**

Alzheimer’s disease (AD), characterized by significant brain volume reduction, is influenced by genetic predispositions related to brain volumetric phenotypes. While genome‐wide association studies (GWASs) have linked brain imaging‐derived phenotypes (IDPs) with AD, existing polygenic risk scores (PRSs) based models inadequately capture this relationship. We develop BrainNetScore, a network‐based model enhancing AD risk prediction by integrating genetic associations between multiple brain IDPs and AD incidence.

**Method:**

Utilizing UK Biobank GWAS summary statistics, we constructed a brain connectivity network from 96 regional brain volume IDPs. This network was expanded into a heterogenous BrainNet graph, incorporating 96 IDPs and 12,043 common variants (SNPs) linked to each IDP. Individual genotype data from independent cohorts, including the Alzheimer’s Disease Neuroimaging Initiative (ADNI), were analyzed. Label propagation algorithms generated individualized predicted scores for IDPs, subsequently aggregated into BrainNetScore via logistic regression.

**Result:**

BrainNet was built from GWAS summary statistics of 96 brain volume IDPs and individual genotype data for 914 samples (550 AD cases, 364 cognitive normal controls) from ADNI. We compared the predictive performance of BrainNetScore against conventional PRS models (Figure 1). The combined PRS + BrainNetScore model showed a superior average AUC of 0.684 ± 0.034, over PRS only (0.595 ± 0.075) and BrainNetScore only (0.666 ± 0.029) models. Including sex as a covariate and APOE genotypes further enhanced predictive accuracy (0.778 ± 0.043).

**Conclusion:**

BrainNetScore significantly improves AD risk prediction when combined with PRSs. This highlights the potential of network‐based approaches and comprehensive genetic analysis in developing personalized medicine strategies for AD. Future work should extend this model to diverse populations.